# Low-Temperature Spark Plasma Sintering of ZrW_2−*x*_Mo*_x_*O_8_ Exhibiting Controllable Negative Thermal Expansion

**DOI:** 10.3390/ma11091582

**Published:** 2018-09-01

**Authors:** Hui Wei, Marin Hasegawa, Shunsuke Mizutani, Akihisa Aimi, Kenjiro Fujimoto, Keishi Nishio

**Affiliations:** 1 Department of Materials Science and Technology, Tokyo University of Science, Tokyo 1258585, Japan; mcdefgahcm@gmail.com (M.H.); yoku_rkz@yahoo.co.jp (S.M.); k-nishio@rs.noda.tus.ac.jp (K.N.); 2 Department of Pure and Applied Chemistry, Tokyo University of Science, Chiba 2788510, Japan; akihisa.aimi@rs.tus.ac.jp (A.A.); fujimoto_kenjiro@rs.tus.ac.jp (K.F.)

**Keywords:** ZrW_2−*x*_Mo*_x_*O_8_, spark plasma sintering, dense sintered body, thermal analysis, negative thermal expansion

## Abstract

Molybdenum-doped zirconium tungstate (ZrW_2−*x*_Mo*_x_*O_8_) has been widely studied because of its large isotropic coefficient of negative thermal expansion (NTE). However, low density and poor sinterability limit its production and application. In this study, relative density greater than 90% single-phase ZrW_2−*x*_Mo*_x_*O_8_ (0.0 ≤ *x* ≤ 1.0) sintered bodies were fabricated by spark plasma sintering (500–600 °C for 10 min) using ZrW_2−*x*_Mo*_x_*O_7_(OH)_2_·2H_2_O precursor powders as the starting material. High-temperature X-ray diffraction and thermomechanical analysis were used to investigate the change in the order–disorder phase transition temperature of the sintered materials; it gradually dropped from 170 °C at *x* = 0.0 to 78 °C at *x* = 0.5, and then to below room temperature at *x* ≥ 0.7. In addition, all sintered bodies exhibited NTE behavior. The NTE coefficient was controllable by changing the *x* value as follows: from −7.85 × 10^−6^ °C^−1^ (*x* = 0) to −9.01 × 10^−6^ °C^−1^ (*x* = 0.6) and from −3.22 × 10^−6^ °C^−1^ (*x* = 0) to −2.50 × 10^−6^ °C^−1^ (*x* = 1.0) before and after the phase transition, respectively. Rietveld structure refinement results indicate that the change in the NTE coefficient can be straightforwardly traced to the thermodynamic instability of the terminal oxygen atoms, which only have one coordination.

## 1. Introduction

Negative thermal expansion (NTE) materials, which exhibit volume contraction upon warming, have been extensively investigated because of their potential utility in many fields, especially in those requiring precisely controllable negative, positive, or near-zero coefficients of thermal expansion composites [1,2,3,4,5,6]. A series of NTE materials, including the AM_2_O_7_, AM_2_O_8_, and A_2_M_3_O_12_ families have been investigated [7,8,9,10,11,12,13,14,15,16,17,18,19]. Among these families, the most representative material is ZrW_2_O_8_, which is widely known for its large NTE coefficient (−4.9 × 10^−6^ °C^−1^ to −8.8 × 10^−6^ °C^−1^) over a wide temperature range (−271 °C to 777 °C) and its three-dimensional isotropic (cubic crystal) structure [7,8]. The NTE mechanism of this material is still being discussed; the rigid unit mode (RUM) model proposed by Evans et al. is the most widely accepted [8,9]. The basic model for the NTE mechanism is based on the idea that the rotation of linked polyhedra pull them in toward each other. This motion is seen more locally as the flexing of the Zr–O–W linkage through transverse motions of the O atoms without significant stretching of the Zr–O and W–O bonds.

Another special thermal property of ZrW_2_O_8_ is its order–disorder phase transition; the change from a low-temperature thermally stable cubic phase (α-ZrW_2_O_8_, space group: P 2_1_ 3) to a high-temperature one (β-ZrW_2_O_8_, space group: P a $\bar{3})$ at around 170 °C. This nearly halves the NTE coefficient, from −8.8 × 10^–6^ °C^−1^ to −4.9 × 10^−6^ °C^−1^ [8]. This abrupt change in the coefficient is a disadvantage in practical applications if the phase transition temperature of the material is within the working temperature range. Previous studies found that the reason for this phase transition is related to the breaking of the free W–O_terminal_ bonds (terminal oxygen atoms have only one coordination) in the framework structure. In this sense, it is clear that substitution of the W^6+^ cation within the crystal structure by another cation strongly affects the phase transition temperature. Because Mo^6+^ and W^6+^ have the same coordination number and the values of the ionic radius and electronegativity are very close, the phase transition temperature of ZrW_2_O_8_ can be adjusted by partially doping Mo^6+^ in W^6+^. Several studies have shown that increasing Mo substitution in ZrW_2−*x*_Mo*_x_*O_8_ leads to a lower phase transition temperature [11,20,21,22,23]. Evans et al. reported that the phase transition temperature shifted to below room temperature when *x* = 1, meaning that the discontinuity of NTE is effectively removed at ambient temperature [11,20].

The vast majority of research using X-ray diffraction analysis and the differential scanning calorimetry method to determine the NTE coefficient and the phase transition temperature of ZrW_2−*x*_Mo*_x_*O_8_ powders. However, there was little research about the fabrication of ZrW_2−*x*_Mo*_x_*O_8_ sintered bodies and evaluation of its thermal expansion properties. In terms of application, the fabrication of ZrW_2−*x*_Mo*_x_*O_8_ sintered bodies and the control of their thermal expansion properties are particularly important.

To our knowledge, it is difficult to obtain a pure, dense sintered body using a high-temperature solid-phase method because of the poor sinterability and high-temperature thermodynamically metastable property of ZrW_2−*x*_Mo*_x_*O_8_. We succeeded in fabricating undoped ZrW_2_O_8_ sintered bodies with a relative density of 95% by spark plasma sintering (SPS) at a relatively low-temperature (600 °C) [24].

In this paper, we describe the fabrication of dense, pure ZrW_2−*x*_Mo*_x_*O_8_ (0.0 ≤ *x* ≤ 1.0) sintered bodies and the effect of Mo substitution for W on the phase transition temperature and NTE coefficients.

## 2. Materials and Methods

The ZrW_2−*x*_Mo*_x_*O_8_ sintered bodies were fabricated by SPS using ZrW_2−*x*_Mo*_x_*O_7_(OH)_2_·2H_2_O precursor powders as the starting material. The precursor powders were prepared by a sol-gel method combined with a hydrothermal process. ZrOCl_2_·8H_2_O (Kishida Chemical, Osaka, Japan, 99%) was dissolved in a mixture solvent consisting of 4 mol/L acetic acid and 2-butanol in air. WCl_6_ (Kojundo Chemical, Saitama, Japan, 99.99%) and MoCl_5_ (Sigma Aldrich, St. Louis, MO, USA, 95%) were dissolved in ethanol in accordance with the stoichiometric ratio of W/Mo = (2 − *x*):*x* (*x* = 0.0, 0.2, 0.5, 0.6, 0.7, 1.0) in a nitrogen atmosphere, and these solutions were mixed and stirred. The mixed solution of W and Mo was poured into the Zr solution and then stirred for 72 h at room temperature. The dry gel powders (sol-gel precursor powders) were prepared by heating the mixed solution of W, Mo, and Zr at 80 °C in the silicone oil bath. In order to improve the crystallinity of the sol-gel precursor powders, the dry gel powders were dispersed in distilled water, placed in a Teflon-lined Parr bomb, and heated with a mantle heater at 180 °C for 18 h. The synthesized powders were dried in an oven at 60 °C, producing a crystalline ZrW_2−*x*_Mo*_x_*O_7_(OH)_2_·2H_2_O precursor. Subsequently, ZrW_2−*x*_Mo*_x_*O_7_(OH)_2_·2H_2_O precursor powders were pre-calcined at 300–450 °C in an oven. About 2 g pre-calcined powders were loaded into graphite dies with an inner diameter of 10 mm and then pressed at an applied pressure of 50 MPa. The applied pressure was held constant until the end of the sintering period. The compacts were sintered at 500–600 °C for 10 min at 100 °C/min in argon atmosphere using an SPS apparatus (SPS-515S; Fuji Electronic Industrial, Saitama, Japan), with a pulse duration of 3.3 ms and on/off pulse intervals of 12:2 as recommended by the manufacturer. In this study, each sintered body was repetitively fabricated more than twice under the same conditions.

The relative densities of the ZrW_2−*x*_Mo*_x_*O_8_ sintered bodies were measured using Archimedes’ principle with distilled water. The density of distilled water is 0.99754 g/cm^3^ at a room temperature of 23 °C. The microstructural characteristics of the sintered bodies were examined with a scanning electron microscope (SEM) equipped with an X-ray energy-dispersive spectrometer (EDS) (JSM-IT100; JEOL, Tokyo, Japan). Phase identification was carried out using an X-ray powder diffraction (XRD) diffractometer (Ultima IV; Rigaku, Tokyo, Japan). The XRD measurements were carried out in a range of 10° ≤ 2θ ≤ 80° at a scan speed of 6°/min, at 45 kV/40 mA, and with Cu–Kα radiation (λ = 0.15406 nm). The NTE coefficients of the samples were measured with a thermodynamic analyzer (TMA-60/60H; Shimadzu, Kyoto, Japan). The data were collected at a heating rate of 1 °C/min from room temperature to 400 °C.

To investigate the change in the phase transition temperature and NTE coefficient, high-temperature XRD (HT-XRD) experiments and Rietveld structure refinement were utilized. HT-XRD was performed using an X’Pert PRO X-ray diffractometer (Empyrean; PANalytical, Almelo, The Netherlands) with a semiconductor detector and a high-temperature attachment (HTK1200). Rietveld structure refinement was performed using General Structure Analysis System (GSAS) software (Los Alamos National Laboratory, Los Alamos, NM, USA) [25]. The Rietveld method was used at each temperature to refine the lattice parameter, the fractional coordinates, the overall temperature factor, the histogram scale factor, the background terms, and the pseudo-Voigt peak shape parameters. The visualization for electronic and structural analysis (VESTA) program was used for drawing the crystal structures [26].

## 3. Results and Discussion

### 3.1. Fabrication of Dense ZrW_2−x_Mo_x_O_8_ Sintered Bodies

**Figure 1:**
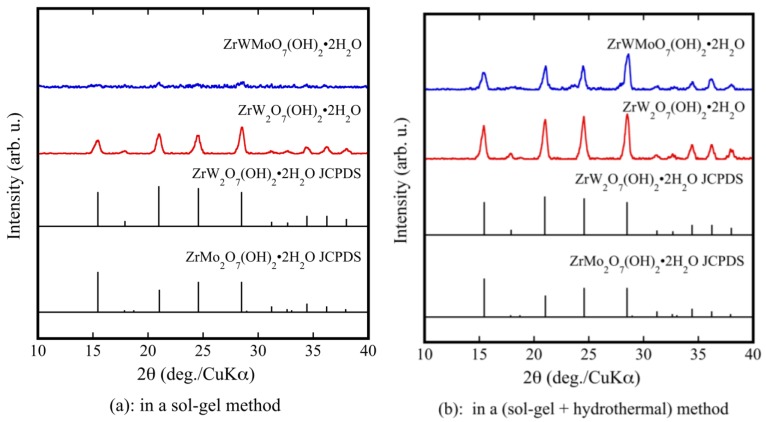

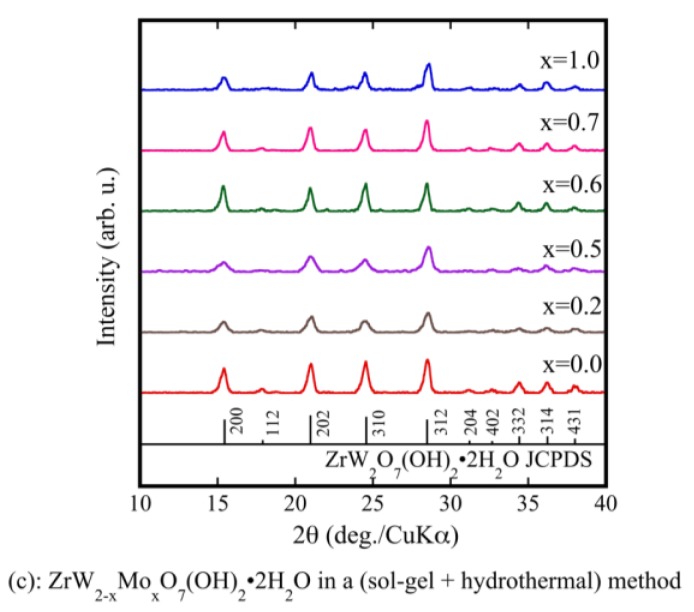
X-ray powder diffraction (XRD) patterns of ZrW_2−*x*_Mo*_x_*O_7_(OH)_2_·2H_2_O precursor powders in a sol-gel method (**a**); and a sol-gel method combines with hydrothermal method (**b**,**c**).

Figure 1a shows the XRD patterns of ZrW_2−*x*_Mo*_x_*O_7_(OH)_2_·2H_2_O (*x* = 0, 1) precursor powders prepared using only a sol-gel method. Although pure ZrW_2_O_7_(OH)_2_·2H_2_O could be synthesized, ZrWMoO_7_(OH)_2_·2H_2_O could not be synthesized. The prepared powders were subjected to a hydrothermal process. As shown by the XRD results in Figure 1b, the precursors were well crystallized. They were indexed as pure ZrW_2_O_7_(OH)_2_·2H_2_O and ZrWMoO_7_(OH)_2_·2H_2_O. The fine precursor powders obtained using the sol-gel method combined with the hydrothermal process had higher crystallinity than those prepared using only the sol-gel method. Figure 1c shows the XRD patterns of the ZrW_2−*x*_Mo*_x_*O_7_(OH)_2_·2H_2_O (0.0 ≤ *x* ≤ 1) precursors. It can be observed that the precursors were well crystallized and were indexed as pure ZrW_2_O_7_(OH)_2_·2H_2_O without any detectable impurities.

**Figure 2:**
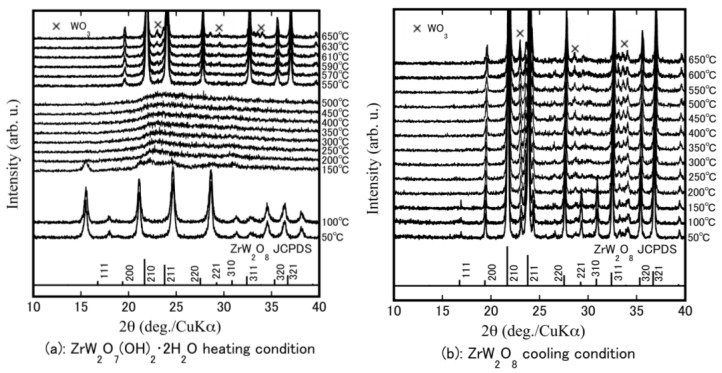
High temperature (HT)-XRD patterns of Zr–W–O system precursor at a temperature program of heating from 50 °C to 650 °C (**a**); and then cooling from 650 °C to 50 °C (**b**).

HT-XRD was performed to determine at which temperatures structural changes occur and to investigate the formation temperature of ZrW_2−*x*_Mo*_x_*O_8_. Figure 2a shows the rising temperature results for the ZrW_2_O_7_(OH)_2_·2H_2_O precursor powders. The precursor phase remained stable from room temperature to 100 °C. It can be observed that the crystalline precursors became amorphous at 200–500 °C. This change in the state is attributed to removal of the hydrate water in the crystalline precursor. High-temperature phase β-ZrW_2_O_8_ was obtained by heating at 550 °C. The β-ZrW_2_O_8_ was stable up to 630 °C; a small amount of WO_3_ as an impurity was generated at temperatures above 630 °C. Figure 2b shows the falling temperature results. The peak intensities of the 111, 221, and 310 reflections increased as the temperature was reduced from 200 °C to 150 °C. The high-temperature β-phase changed to the low-temperature stable phase (α-ZrW_2_O_8_). The 111, 221, and 310 reflections are attributed to the α-ZrW_2_O_8_. The reflection peaks shifted to a higher angle (2θ degree) as the temperature was reduced, meaning that the volume of material expanded during cooling.

**Figure 3:**
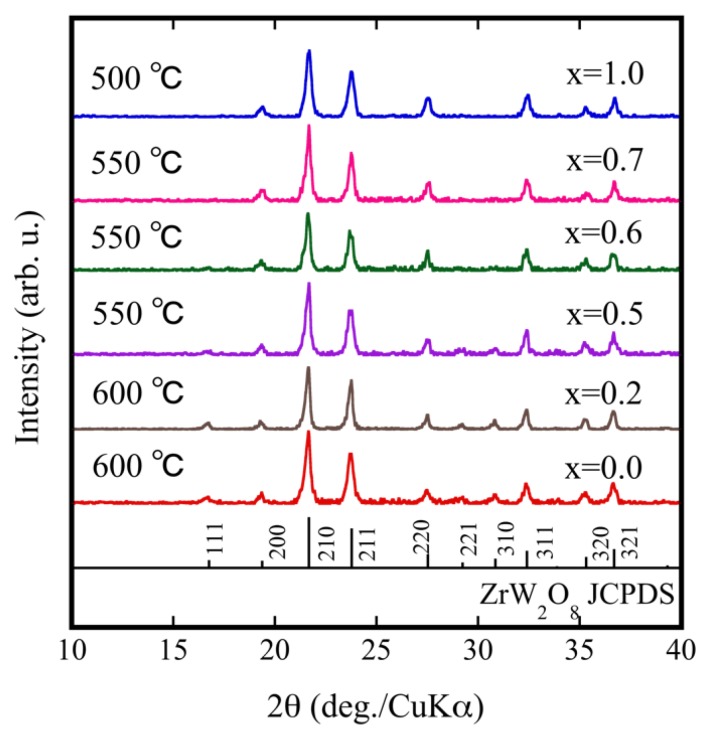
XRD patterns of ZrW_2−*x*_Mo*_x_*O_8_ sintered bodies.

Using the results shown in Figure 2a, we performed multiple temperature comparison experiments to fabricate ZrW_2−*x*_Mo*_x_*O_8_ sintered bodies by SPS. The optimum sintering temperature for each sample depended on various factors (relative density, crystallinity, etc.). Figure 3 shows the XRD patterns for each ZrW_2−*x*_Mo*_x_*O_8_ sintered body under optimal sintering temperature conditions. The optimal sintering temperature depended on the Mo content: 600 °C for *x* = 0.0, 550 °C for *x* = 0.5, and 500 °C for *x* = 1.0. The ZrW_2−*x*_Mo*_x_*O_8_ sintered bodies were pure phase and consistent with ZrW_2_O_8_. The 111, 221, and 310 reflections progressively diminished with increasing Mo substitution and almost disappeared when *x* ≥ 0.7. The reflection intensity decreased in the order of 111, 221, and 310 in accordance with the generation of β phase and substitution of W by Mo. These results suggest that the crystal phase of the ZrW_2−*x*_Mo*_x_*O_8_ compound depends on the *x* value. When *x* was increased to 0.7 at room temperature, ZrW_2−*x*_Mo*_x_*O_8_ completely converted to the β phase. The density of the obtained ZrW_2−*x*_Mo*_x_*O_8_ sintered bodies and theoretical density are shown in Table 1. All sintered bodies with relative densities of more than 90% were obtained with a shorter heat treatment compared with the conventional sintering process. Thus, SPS is a promising method for densification of body structures as it promotes fast heating and fast cooling of sintered bodies.

**Figure 4:**
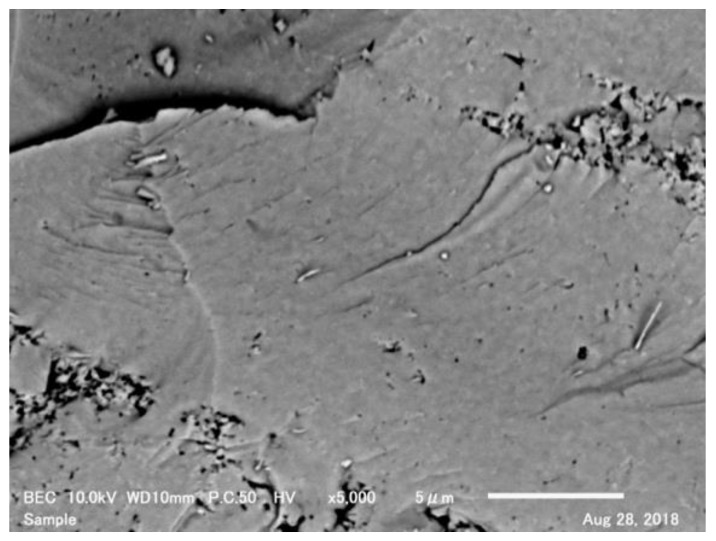
Scanning electron microscope (SEM) image of fractured surface of ZrW_2_O_8_ sintered body.

Figure 4 shows SEM images of the fractured surface of the sintered body. The SEM image shows that the sintered body was very dense. This is attributed to the applied pressure during SPS, which enhanced densification over grain-growth-promoting diffusion mechanisms. In addition, EDS point analysis of the fractured surface was performed to determine the composition of the black areas. A comparison of the elemental compositions of the black and bright areas revealed no significant change in the Zr, W, and O contents, indicating that the black areas were not impure substances.

### 3.2. Evaluation of Thermal Expansion Properties of ZrW_2−x_Mo_x_O_8_ Sintered Bodies

**Figure 5:**
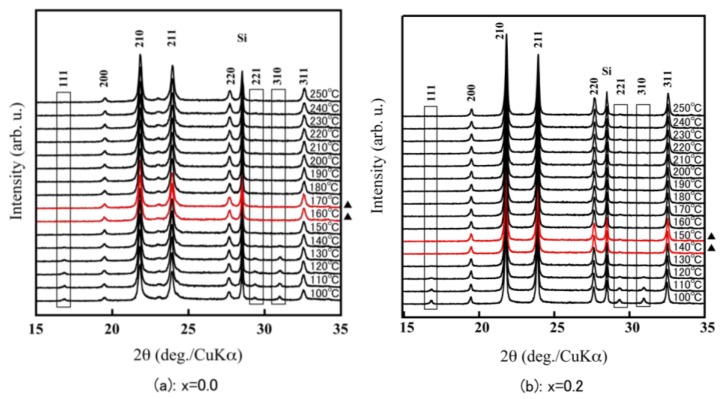

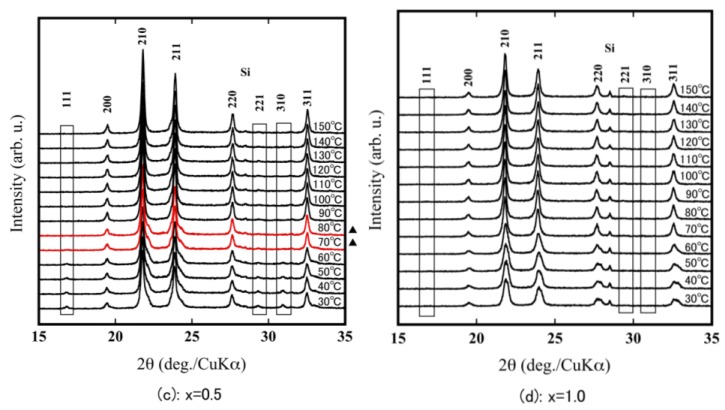
HT-XRD patterns of ZrW_2−*x*_Mo*_x_*O_8_ sintered bodies (**a**): *x* = 0.0; (**b**): *x* = 0.2; (**c**): *x* = 0.5; (**d**): *x* = 1.0; (▲: phase transition interval).

For the α-ZrW_2−*x*_Mo*_x_*O_8_, the 111 reflection in the vicinity of 17° and the 221 and 310 reflections around 30° were clearly observed at room temperature. In contrast, for the β-ZrW_2−*x*_Mo*_x_*O_8_, there were no reflections around 17° and 30°. Therefore, because of the existence of the 111, 221, and 310 reflections in the X-ray diffraction pattern, the phase transition temperature could be calculated from α phase to β phase from the high-temperature X-ray diffraction patterns (Figure 5). The phase transition temperature of ZrW_2_O_8_ corresponded to the previously reported temperature (around 160 °C–170 °C), that for *x* = 0.2 was slightly lower (140 °C–150 °C), and that for *x* = 0.5 was 70 °C–80 °C. These findings show that the phase transition temperature of ZrW_2−*x*_Mo*_x_*O_8_ decreases with increasing Mo substitution. The disappearance of the 111, 221, and 310 reflections in the XRD pattern of ZrWMoO_8_ indicates that ZrWMoO_8_ exists in the β phase above room temperature.

**Figure 6:**
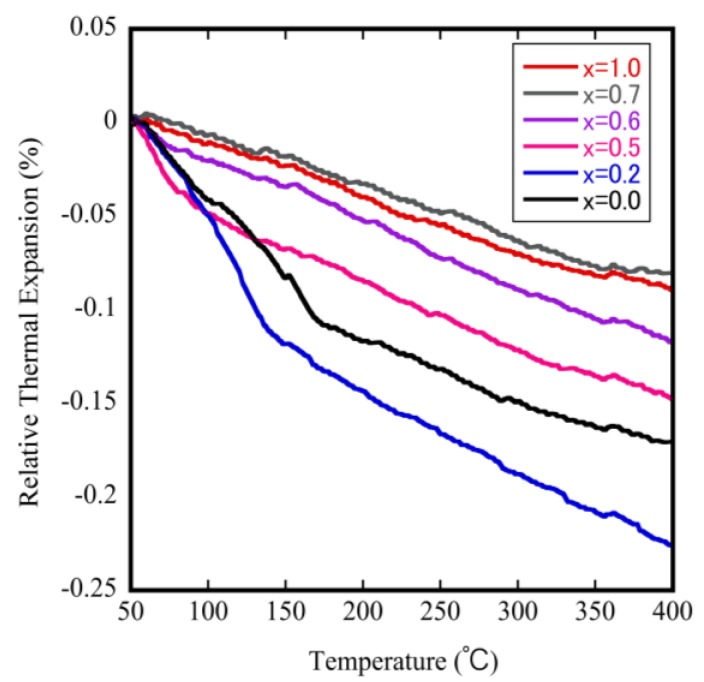
Thermodynamic analyzer (TMA) results for ZrW_2−*x*_Mo*_x_*O_8_ sintered bodies.

To investigate and identify the effect of Mo doping on the phase transition temperature and on the NTE coefficients, we measured the relative thermal expansion of ZrW_2−*x*_Mo*_x_*O_8_ sintered bodies as a function of temperature. The results are shown in Figure 6. The downward trend of each curve with increasing temperature reflects the NTE property. The bending point in each curve is attributed to a change in the NTE coefficient, which corresponds to the occurrence of phase transitions in this study. These results show that the phase transition shifts to the lower temperature side as the Mo content is increased. The phase transition from α-phase to β-phase could be investigated when *x* ≤ 0.6, but not when 0.7 ≤ *x* within the measurement temperature range. The decrease in the phase transition temperature is attributed to the fact that the electronegativity of Mo (2.16) is less than that of W (2.36). This means that when the Mo content is gradually increased, the separation energy of W(Mo)–O gradually decreases, leading to a reduction in the phase transition temperature.

The NTE coefficient can be automatically calculated from the change in the dimensions of the material with temperature. The values for the ZrW_2−*x*_Mo*_x_*O_8_ samples are shown in Table 2. All samples showed the NTE property: the NTE coefficient can be controlled by changing the *x* value as follows: from −7.85 × 10^−6^ °C^−1^ (*x* = 0) to −9.01 × 10^−6^ °C^−1^ (*x* = 0.6) and from −3.22 × 10^−6^ °C^−1^ (*x* = 0) to −2.50 × 10^−6^ °C^−1^ (*x* = 1.0) before and after the phase transition, respectively. The NTE coefficients of the β phase decreased as more Mo was substituted. The reason for this phenomenon will be discussed below.

### 3.3. Rietveld Structure Refinement of ZrW_2−x_Mo_x_O_8_ Compounds

Crystal structures of undoped and Mo-substituted ZrW_2_O_8_ (ZrW_2_O_8_, ZrW_1.8_Mo_0.2_O_8_, ZrW_1.5_Mo_0.5_O_8_) compounds were generated using refined lattice parameters and atomic coordinates at room temperature, 100 °C, 150 °C, 200 °C, 250 °C, and 300 °C. The structural parameters obtained from Rietveld refinement of these samples at room temperature and 300 °C are listed in Table 3. Comparison of these samples revealed no obvious changes in the fractional coordinates with changes in the temperature or Mo content, indicating that the addition of Mo does not change the original structure of ZrW_2_O_8_.

**Figure 7:**
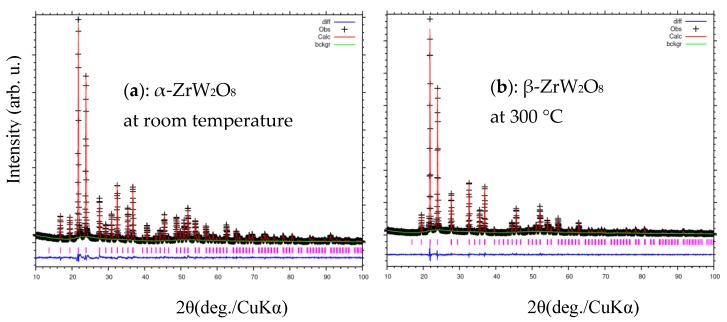
Rietveld refinement of X-ray diffraction patterns of ZrW_2_O_8_ at (**a**) room temperature; and (**b**) 300 °C.

**Figure 8:**
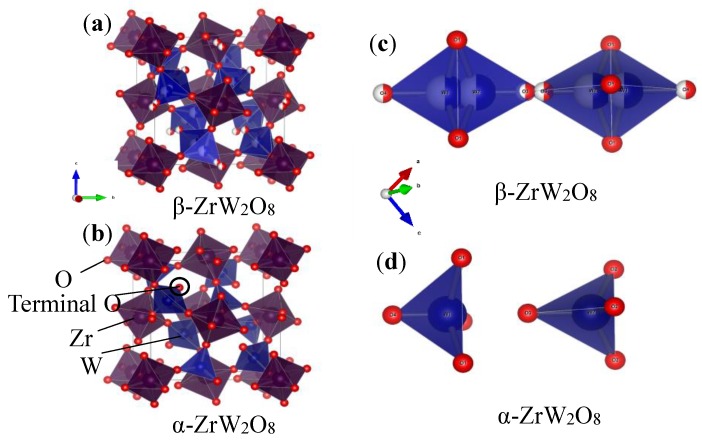
Crystal structure of (**a**) α phase and (**b**) β phase of ZrW_2_O_8_; WO_4_ groups in <111> direction of (**c**) α phase and (**d**) β phase of ZrW_2_O_8_.

Figure 7 and Figure 8a,b shown the refinement results and corresponding crystal structures of ZrW_2_O_8_ at room temperature for α phase and at 300 °C for β phase. The crystal structures contain corner-sharing ZrO_6_ octahedra and WO_4_ tetrahedra. The ZrO_6_ octahedra share all six corners with the WO_4_ tetrahedra, whereas the WO_4_ tetrahedra share only three of their four corners with the ZrO_6_ octahedra. In other words, each WO_4_ tetrahedron has one coordination oxygen atom, which is called ‘terminal’ oxygen. (In this study, O4 and O3 correspond to the terminal oxygen in the W1O_4_ and W2O_4_ tetrahedra, respectively). The NTE property of both phases of the ZrW_2_O_8_ can be simply defined as follows: the WO_4_ polyhedra rotate inward as the temperature rises, and the ZrO_6_ octahedra stretch to shrink the decrease in volume due to this rotation^2^. The only structural difference between the α and β phases is in the W1O_4_ and W2O_4_ tetrahedra. A view of the WO_4_ groups in the <111> direction of ZrW_2_O_8_ at RT and 300 °C is shown in Figure 8c,d. The WO_4_ tetrahedra are arranged in pairs along the main three-fold axis of the cubic unit cell, forming condensed W_2_O_8_ units. The ‘terminal’ O atom of each WO_4_ tetrahedron points in the same direction in the W_2_O_8_ unit, leading to an O4–W1···O3–W2 arrangement. In the α phase structure, the two WO_4_ tetrahedra are not bound together at a certain distance. In comparison, the inversion of W1O_4_ and W2O_4_ tetrahedra is arranged in pairs, forming condensed W_2_O_8_ polyhedra when the phase transition occurs.

**Figure 9:**
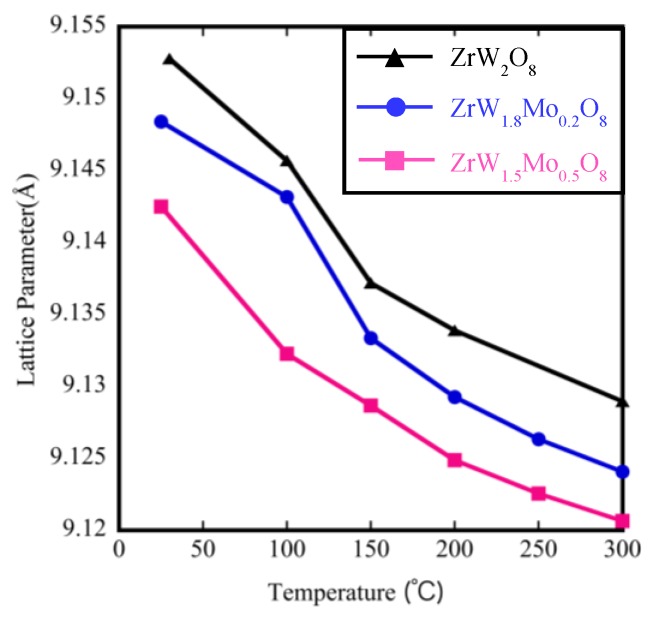
Lattice parameters of ZrW_2−*x*_Mo*_x_*O_8_.

The temperature dependence of the lattice parameters on the Mo content derived from Rietveld refinement is shown in Figure 9. The lattice parameter for these samples clearly decreased with the increase in Mo content at the same temperature, which is reasonable given the smaller radius of Mo^6+^ (0.41 Å) compared with that of W^6+^ (0.42 Å). In addition, the lattice parameters of all samples contracted linearly with increasing temperature, indicating that the thermal expansion behavior is negative. The coefficient of thermal expansion calculated on the basis of the relationship between the temperature and lattice parameters is shown in Table 4. The NTE coefficients changed from −9.88 × 10^−6^ °C^−1^ to −5.35 × 10^−6^ °C^−1^ for ZrW_2_O_8_. The reduction in the β phase with the addition of Mo content is consistent with the decreasing trends of the TMA results (Table 2). We compared the NTE coefficients in Table 2 and Table 4 and found that the results of the two measurements differed. The reason for this difference is attributed to two factors. The first one is the presence of pores inside the sintered body, which could be roughly observed by SEM. The second one is the presence of systematic errors due to the use of different instruments, temperature controllers, and high-temperature furnaces.

**Figure 10:**
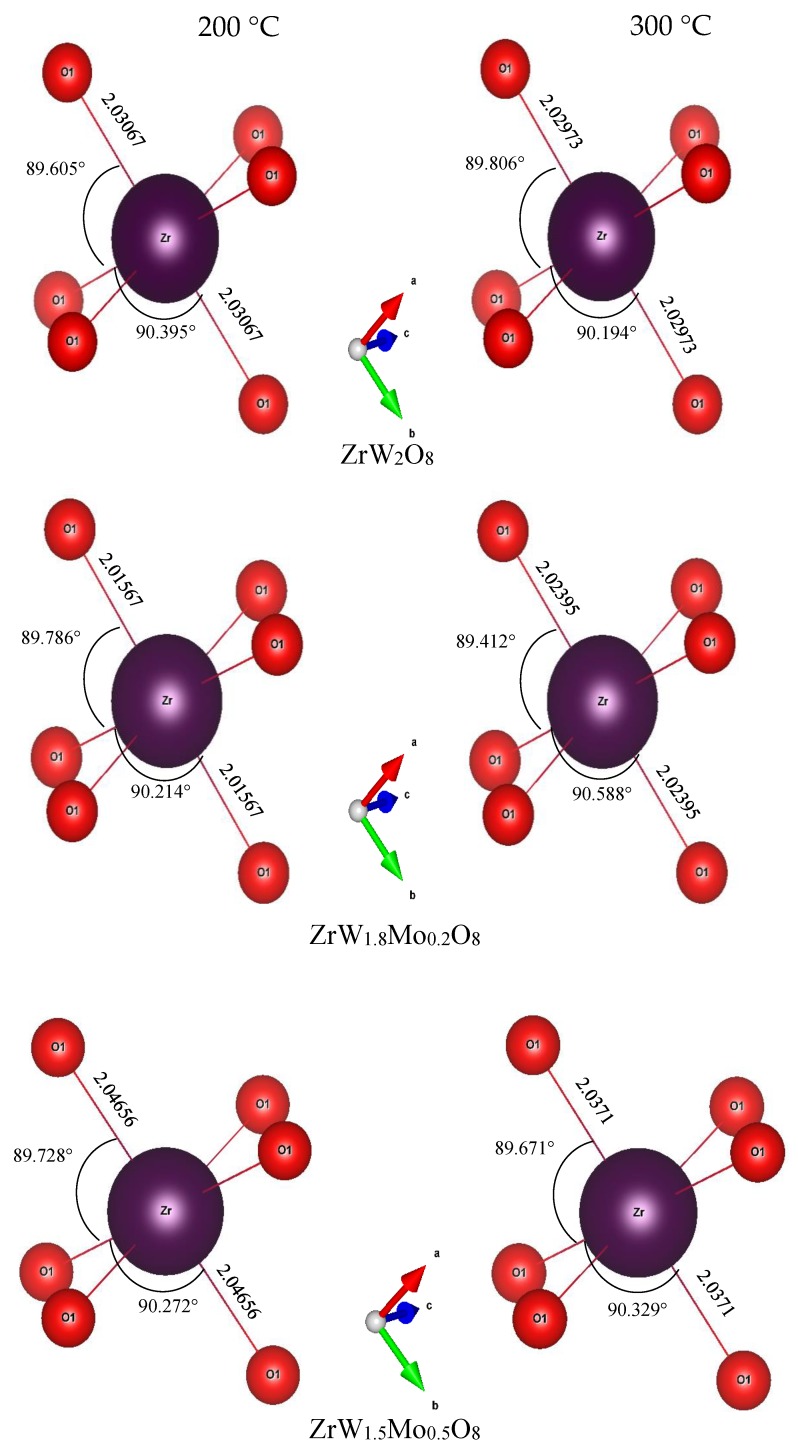
Bond lengths and bond angles of ZrO_6_ octahedron in ZrW_2−*x*_Mo*_x_*O_8_ compounds.

**Figure 11:**
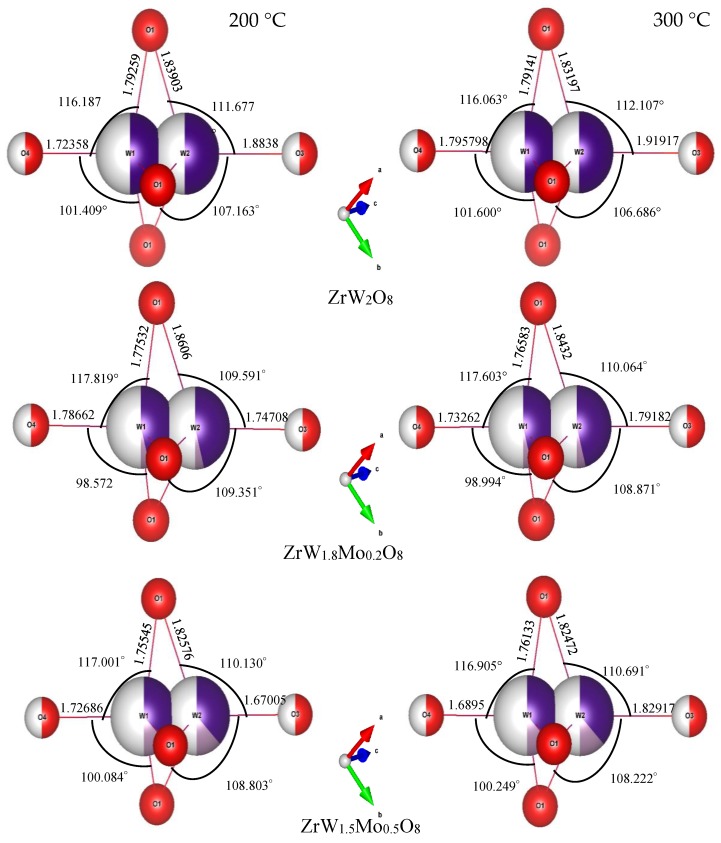
Bond lengths and bond angles of W(Mo)_2_O_8_ polyhedra in ZrW_2−*x*_Mo*_x_*O_8_ compounds.

To intuitively investigate the mechanism of the reduction in the NTE coefficients in the β phase with an increase in Mo content, we examined the variations in bond length, bond angle, and distortion of ZrO_6_ and WO_4_ polyhedra. The bond lengths and angles of ZrO_6_ and WO_4_ polyhedra for all samples at 200 °C and 300 °C are shown in Figure 10 and Figure 11. For the ZrO_6_ octahedra, the Zr–O1 bond lengths in each polyhedron are equal, and the Mn–O bond length is close to the sum of the ionic radii (r_Zr_ + r_O_ = 2.07 Å). The variations in bond length were minor; the lengths varied from 2.01 Å to 2.04 Å with changes in the Mo content. The bond angles of O1–Zr–O1 exhibited minor deviations from the octahedral symmetry, while the bond angle of O1–Zr–O1 remained constant at around 90°, indicating that the ZrO_6_ octahedra in all samples were close to a regular octahedron. This means that the ZrO_6_ octahedra in all samples resembled a rigid body.

Compared with the rigid ZrO_6_ octahedra, the W(Mo)_2_O_8_ polyhedra appear to be distorted to a large extent. In these compounds, there were three identical W(Mo)1–O1 bonds and three identical W(Mo)2–O1 bonds, while the W1–O4 and W2–O3 bonds existed alone. The angles of each bond in the W(Mo)_2_O_8_ polyhedra did not change with a change in Mo content—they were basically fixed at 116(1)° for O1–W(Mo)1–O1, 109(1)° for O1–W(Mo)2–O1, 99(2)° for O1–W(Mo)1–O4, and 108(1)° for O1–W(Mo)2–O3. The bond lengths for W(Mo)1–O1 decreased with an increase in Mo content, which may be related to the fact that the ionic radius of Mo^6+^ is less than that of W^6+^.

**Figure 12:**
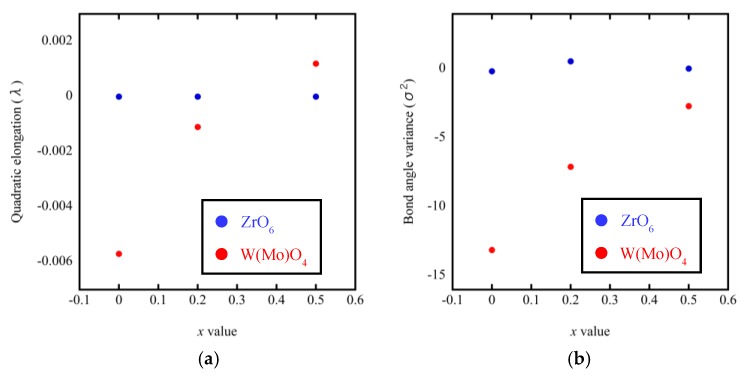
Quadratic elongation (**a**) and bond angle variance (**b**) of ZrO_6_ and W(Mo)_2_O_8_ polyhedra in ZrW_2−*x*_Mo*_x_*O_8_.

A noteworthy finding is that the bond lengths of W(Mo)1–O4 and W(Mo)2–O3 not only varied greatly with the temperature (from 200 °C to 300 °C), but also varied greatly with the Mo content. This may be related to the one-coordination of O_3_ and O_4_ atoms. Different W(Mo)–O bond lengths in different directions indicate stronger distortion in the polyhedra. Two factors necessary for distortion of a polyhedron are quadratic elongation (λ) and bond angle variance (σ^2^) [27]. The variance values of the distortion constants λ and σ^2^ between 200 °C and 300 °C are shown in Figure 12. We can see that the ZrO_6_ octahedron is basically in an undistorted state, which is attributed to all bond lengths Zr–O1 being equal and all bond angles being close to 90°. In the W(Mo)_2_O_8_ polyhedron, the degree of distortion increased greatly with the temperature because of the instability of the one-coordinate oxygen atoms. The degree of distortion gradually decreased with increasing Mo content, resulting in a decrease in the NTE coefficient.

## 4. Conclusions

Single-phase ZrW_2−*x*_Mo*_x_*O_8_ (0 ≤ *x* ≤ 1) sintered bodies with a relative density greater than 90% were obtained by spark plasma sintering using ZrW_2−*x*_Mo*_x_*O_7_(OH)_2_·2H_2_O precursor powders as the starting material and a relatively low-temperature heat treatment (500 °C–600 °C). The α–β phase transition temperature of the ZrW_2−*x*_Mo*_x_*O_8_ sintered bodies decreased as more Mo was substituted at W sites. When *x* ≥ 0.7, the α–β phase transition could not be observed above room temperature. The coefficient of negative thermal expansion of ZrW_2−*x*_Mo*_x_*O_8_ can be controlled by changing the amount of Mo substituted at W sites. These single ZrW_2−*x*_Mo*_x_*O_8_ (0 ≤ *x* ≤ 1) sintered bodies with a controllable coefficient of negative thermal expansion have a wide range of applications.

## Figures and Tables

**Table 1:** Measurement density and relative densities of ZrW_2−*x*_Mo*_x_*O_8_ sintered bodies.

Compound	*x* = 0.0	*x* = 0.2	*x* = 0.5	*x* = 0.6	*x* = 0.7	*x* = 1.0
Sintering temperature (°C)	600	600	550	550	550	500
Density (g/cm^3^)	4.6271	4.6832	4.4217	4.4855	4.2760	4.1689
Theoretical density (g/cm^3^)	5.08	4.93	4.71	4.63	4.56	4.33
Relative density (%)	91.0846	94.9939	93.8790	96.8790	93.7719	96.2794

**Table 2:** Negative thermal expansion (NTE) and phase transition temperature for ZrW_2−*x*_Mo*_x_*O_8_ sintered bodies (50–400 ℃) by thermodynamic analyzer (TMA).

Coefficient of Thermal Expansion (×10^–6^ °C^−1^)			Phase Transition Temperature (°C)
Compound	α Phase	β Phase	
*x* = 0.0	−7.85 (50–130 °C)	−3.22 (200–400 °C)	170
*x* = 0.2	−11.79 (50–130 °C)	−4.17 (200–400 °C)	145
*x* = 0.5	−15.34 (50–70 °C)	−3.16 (200–400 °C)	78
*x* = 0.6	−9.01 (50–70 °C)	−3.21 (200–400 °C)	60
*x* = 0.7	-	−2.91 (200–400 °C)	-
*x* = 1.0	-	−2.50 (200–400 °C)	-

**Table 3:** Rietveld refinement of atomic coordinates and occupancy of ZrW_2−*x*_Mo*_x_*O_8_.

Temperature	Room Temperature			300 °C		
Compound	ZrW_2_O_8_	ZrW_1.8_Mo_0.2_O_8_	ZrW_1.5_Mo_0.5_O_8_	ZrW_2_O_8_	ZrW_1.8_Mo_0.2_O_8_	ZrW_1.5_Mo_0.5_O_8_
Goodness-of-fit (*x*^2^)	1.776	6.993	3.983	1.375	2.928	3.194
Space Group	P 2_1_ 3	P 2_1_ 3	P 2_1_ 3	P a $\bar{3}$	P a $\bar{3}$	P a $\bar{3}$
Atom	Zr	Zr	Zr	Zr	Zr	Zr
*x* = *y* = *z*	0.000319	0.004534	0.002891	0.000000	0.000000	0.000000
Occupancy	1	1	1	1	1	1
Atom	W1	W(Mo)1	W(Mo)1	W1	W(Mo)1	W(Mo)1
*x* = *y* = *z*	0.342614	0.343947	0.351651	0.340273	0.339711	0.338886
Occupancy	1	W1:0.9, Mo1:0.1	W1:0.75, Mo1:0.25	0.5	W1:0.45, Mo1:0.05	W1:0.375, Mo1:0.125
Atom	W2	W(Mo)2	W(Mo)2	W2	W(Mo)2	W(Mo)2
*x* = *y* = *z*	0.601688	0.603332	0.608726	0.603304	0.605205	0.606212
Occupancy	1	W2:0.9, Mo2:0.1	W2:0.75, Mo2:0.25	0.5	W2:0.45, Mo2:0.05	W2:0.375, Mo2:0.125
Atom	O1	O1	O1	O1	O1	O1
*x*	0.042345	0.021103	0.008577	0.050445	0.055887	0.05519
*y*	−0.19759	−0.179137	−0.183323	−0.205553	−0.201601	−0.202851
*z*	−0.058376	−0.066893	−0.057469	−0.069239	−0.073105	−0.0735229
Occupancy	1	1	1	1	1	1
Atom	O2	O2	O2	-	-	-
*x*	0.078837	0.094246	0.121337	-	-	-
*y*	−0.057928	−0.071485	−0.094012	-	-	-
*z*	0.211437	0.221976	0.205825	-	-	-
Occupancy	1	1	1	-	-	-
Atom	O3	O3	O3	O3	O3	O3
*x* = *y* = *z*	0.486051	0.479937	0.469176	0.509573	0.510211	0.512884
Occupancy	1	1	1	0.5	0.5	0.5
Atom	O4	O4	O4	O4	O4	O4
*x* = *y* = *z*	0.250373	0.262464	0.262221	0.231004	0.228680	0.231430
Occupancy	1	1	1	0.5	0.5	0.5

**Table 4:** High temperature (HT)-X-ray powder diffraction (XRD) calculated NTE coefficients for ZrW_2−*x*_Mo*_x_*O_8_.

Compound	Calculated Coefficient of Thermal Expansion by Lattice Parameter (×10^−6^ °C^−1^)	
	α Phase	β Phase
ZrW_2_O_8_	−9.88	−5.35
ZrW_1.8_Mo_0.2_O_8_	−7.4	−4.72
ZrW_1.5_Mo_0.5_O_8_	-	−3.95
